# Transplantation of Autologous Minced Bladder Mucosa for a One-Step Reconstruction of a Tissue Engineered Bladder Conduit

**DOI:** 10.1155/2013/212734

**Published:** 2013-10-31

**Authors:** Gisela Reinfeldt Engberg, Johan Lundberg, Clara Ibel Chamorro, Agneta Nordenskjöld, Magdalena Fossum

**Affiliations:** ^1^Department of Women's and Children's Health and Center of Molecular Medicine, Karolinska Institutet, Q3:03 Astrid Lindgren Children's Hospital, 171 76 Stockholm, Sweden; ^2^Pediatric Surgery, Unit of Urology, Astrid Lindgren Children's Hospital, Karolinska University Hospital, 171 76 Stockholm, Sweden; ^3^Department of Clinical Neuroscience, Karolinska Institutet and Department of Neuroradiology, Karolinska University Hospital, 171 76 Stockholm, Sweden

## Abstract

Surgical intervention is sometimes needed to create a conduit from the abdominal wall to the bladder for self-catheterization. We developed a method for tissue engineering a conduit for bladder emptying without *in vitro* cell culturing as a one-step procedure. In a porcine animal model bladder, wall tissue was excised and the mucosa was minced to small particles. The particles were attached to a tube in a 1 : 3 expansion rate with fibrin glue and transplanted back by attaching the tube to the bladder and through the abdominal wall. Sham served as controls. After 4-5 weeks, conduits were assessed in respect to macroscopic and microscopic appearance in 6 pigs. Two pigs underwent radiology before termination. Gross examination revealed a patent conduit with an opening to the bladder. Histology and immunostaining showed a multilayered transitional uroepithelium in all cases. Up to 89% of the luminal surface area was neoepithelialized but with a loose attachment to the submucosa. No epithelium was found in control animals. CT imaging revealed a patent channel that could be used for filling and emptying the bladder. Animals that experienced surgical complications did not form conduits. Minced autologous bladder mucosa can be transplanted around a tubular mold to create a conduit to the urinary bladder without *in vitro* culturing.

## 1. Introduction

Autologous tissue from the urinary system for tissue engineering would be an appealing approach for tissue reconstruction in pediatric urology. Conditions with lack of bladder tissue as well as low compliance and high pressure bladders, often need surgical intervention with an abdominal conduit for self-catheterization and/or a bladder augmenting approach, to reduce the risk of secondary renal failure.

To date, reconstructions in the urinary pathway often use autologous intestinal tissue. Primarily, the appendix is used for self-catheterization of the bladder [1]. If nonexisting, or if the appendix is preferred for the creation of a stoma for antegrade enemas [2], the small intestine can be reconfigured and it can serve as a conduit for catheterization of the bladder [3, 4]. Drawbacks with using intestinal tissue for reconstructive urology include the need for laparotomy and high risk of mucus and stone formations as well as infections [5]. If the use of appendix is contraindicated, removal of distal small intestine can lead to secondary malabsorption of bile acids that induce diarrhea and malabsorption of liposoluble vitamins leading to malnutrition [6, 7].

 For tissue engineering, the algorithm for clinical studies in the urinary pathway and for skin replacements has so far mostly included *in vitro* cell culturing for tissue expansion [8–11]. This procedure is however time consuming, demands high standard laboratory facilities, and is associated with high costs and will demand highly specialized centers.

In previous studies, we transplanted small particles of skin or urothelium by attaching the minced tissue to three-dimensional tubular molds into the subcutaneous tissue in pigs [12, 13]. After withdrawing the mold, a continuous epithelium in the luminal surface of subcutaneous tunnels was formed already after 2 weeks.

 The objective of the present study was to reconstruct such a three-dimensional conduit through the abdominal wall into the bladder in a large animal experimental model in order to catheterize the bladder through the canal.

## 2. Materials and Methods

Eleven 15–25 kg (11-12 weeks old) female Yorkshire-Swedish Country Pigs (Vallrum, Sala, Sweden) were initially used in this study. The protocol was pre-approved by the Stockholm County Committee on Animals and all procedures conformed to the regulations for animal use, as well as relevant federal statutes.

The pigs were stalled in pairs in every box except for a few hours postoperatively when they were kept single until they regained full normal activity. They had full supply of fresh water and were fed according to standard protocols at the animal institution where they were stalled. Heat lamps were used and the ground was covered with straw for rooting and toys for enrichment.

Protocols were held to ensure normal urination pre- and postoperatively. Dressings and wounds were checked at least twice daily postoperatively to ensure early action if signs of infection, hernias, or other unpredictable events. Animals were euthanized at study endpoint up to 5 weeks posttransplantation.

### 2.1. Surgical Procedures

After a 12-hour fasting, an intramuscular injection of azaperone 2 mg/kg (Stresnil, Janssen-Cilag, Pharma, Austria) was administered as premedication. A combination of tiletamine hypochloride 2,5 mg/kg, zolazepam hypochloride 2,5 mg/kg (Zoletil, Virbac, France), and medetomidine 25 micrograms/kg (Domitor, Orion Pharma, Sweden) was then administered for induction of anesthesia together with atropine 25 micrograms/kg (Atropin, Mylan Inc, Canonsburg, PA) as intramuscular injections. Analgesic treatment was initiated peroperatively by administrating buprenorphine 45 micrograms/kg (Temgesic, RB Pharmaceuticals, Great Britain), carprofen 3 mg/kg (Rimadyl, Orion Pharma, Sweden) intravenously and by local injection of lidocaine (Xylocaine, AstraZeneca, Sweden) before skin incisions.

Phenobarbiturate 15 mg/kg (Pentobarbital, APL, Sweden) was given prior endotracheal intubation and general anesthesia was maintained with 0, 8–2% isoflurane (Isoflurane, Baxter, Deerfield, IL). Glucose 25 mg/mL (Baxter, Deerfield, IL) was administered intravenously and body temperature, saturation, blood pressure, and pulse were monitored during the whole procedure.

**Figure 1:**
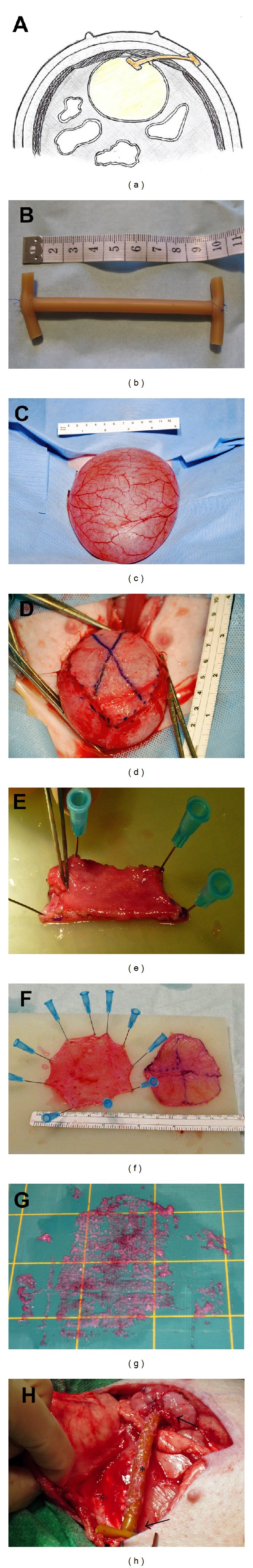
(a) Cartoon demonstrating a cross-section of the pig urinary bladder and abdominal wall with a tube placed to form a conduit from the urinary bladder, through the abdominal muscle wall and to the cutaneous tissue. (b) Tube used for transplantation of minced bladder mucosa showing stopping ends used to hold the tube still. (c) The pig urinary bladder exposed through the surgical wound. (d) Part of the bladder wall being removed. The detrusor muscle has been marked for easy identification and for measuring of tissue biopsy. (e) Mechanical separation of bladder mucosa from the detrusor. (f) Bladder mucosa mounted to the left and separated detrusor to the right. (g) Minced particles of bladder mucosa. (h) Tube with minced particles being placed in the surgical wound. In the top one, the end is coming out from the native bladder (upper arrow). Middle part of tube is resting on the abdominal muscle (asterix) and in the outer end placed under the skin (lower arrow).

With the pig turned to a supine position, the abdominal skin was cleaned with chlorhexidine gluconate (Hibiscrub 40 mg/mL, Regent Medical, England) and with successive applications of chlorhexidine (Klorhexidin 5%, Fresenius Kabi, Sweden). The urinary bladder was catheterized using a 10-French silicone catheter, emptied and then filled with sterile saline solution (approximately 8 mL/kg of body weight). After exposure of the bladder by midline incision below the umbilicus, a lenticular area of the bladder dome measuring 12 cm longitudinally and 9 cm transversally (approximately a forth of the total bladder surface area) was marked, removed, and partly used in this study (Figures 1(c) and 1(d)).

For postoperative analgesia, buprenorphine 45 micrograms/kg (Temgesic, RB Pharmaceuticals, Great Britain) was administered by intramuscular injections in single doses. For infection prophylaxis, an intra-operative dose of trimetoprim 4 mg/kg and sulfonamide 20 mg/kg (Tribrissen vet, Intervet, Sweden) was administered by intramuscular injection and followed by injections twice daily for three days and then once daily for 5 days (total of 9 days).

### 2.2. Mincing the Bladder Mucosa and the Preparation of Tubes

**Figure 2:**
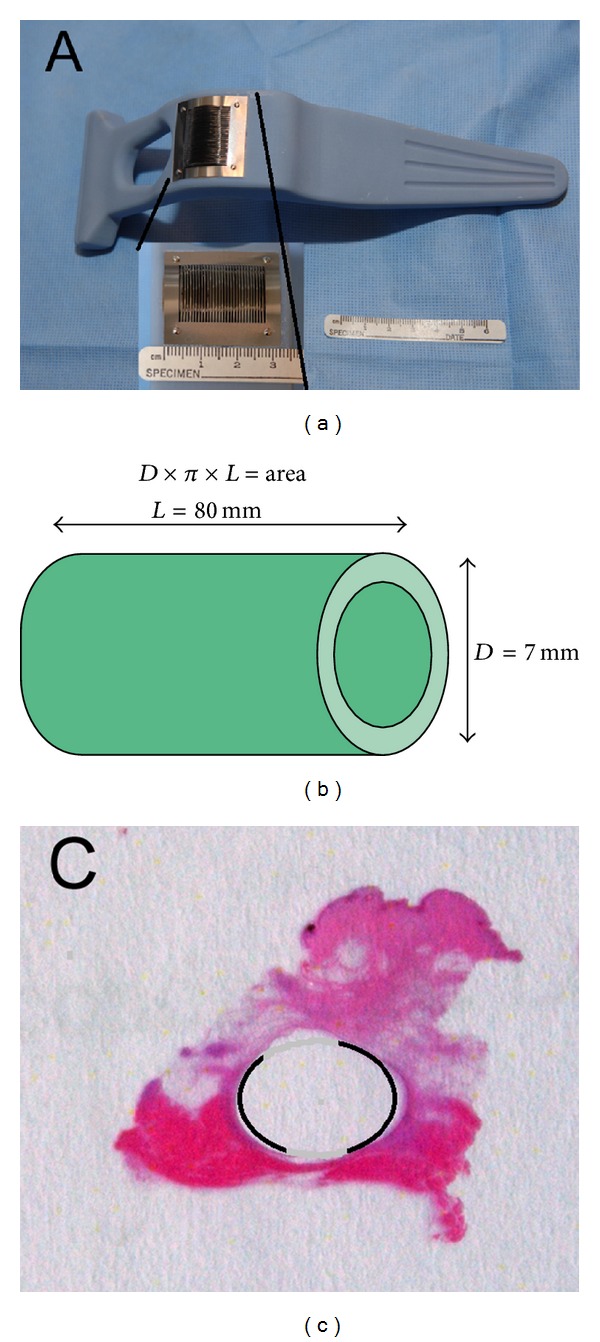
(a) Side view of mincing device and insert demonstrating frontal aspect of central part of device with 30 parallel rotating cutting discs. (b) Formula for calculating area of implanted mold and expected inner area of conduit. (c) Cross-section of conduit on slide with marking of inner surface with epithelial lining. No magnification.

The excised bladder wall was washed in Dulbecco's modified Eagle medium (Sigma-Aldrich, Steinheim, Germany) twice, and the detrusor muscle was removed mechanically with curved scissors for dissection (Figures 1(e) and 1(f)). The mucosal tissue was stretched on a cutting board and thickness was measured with a caliper to approximately 0,2 mm and appropriate amount of bladder mucosa was removed. As we aimed for a 3-fold expansion rate for regeneration of a confluent mucosa *in vivo*, we estimated the area of the inner surface of the conduit by calculating the outer surface of the tubular mold (18 Fr) (Figure 2(b)). Five cm^2^ was therefore separated for use in the study (remaining bladder tissue was used for other studies). Immediately prior to transplantation, the graft was minced with a mincing device that has been developed for this purpose [12, 13] (Figure 2(a)). Briefly, the device cuts with 30 rotating cutting disks placed with 0.8 mm distance from each other. By running the device twice over the tissue in perpendicular directions, small square particles measuring approximately 0.2 × 0.8 × 0.8 mm in size were formed (Figure 1(g)). 

18 Fr latex catheters covered with a silicone elastomer (Bard Foley Catheter, C.A. Bard Inc, Covington, GA) were cut into 10 cm long tubes. Each end was closed with a 3-cm long segment of 16 Fr latex catheters sutured with 4-0 nylon (Ethilon, Ethicon, Johnson & Johnson, Sweden) at right angles (stopping end) in order to secure the tubes into the bladder and to lower the risk of movement in the subcutaneous tissue (Figures 1(a) and 1(b)). To minimize the risk of infection during take of the transplants, the conduit was blocked until sedation for radiology studies and termination. 

### 2.3. Transplantation

The 8 cm long outer surface (14.5 cm^2^) of the tube was covered with two thin layers of the sealer protein concentrate (fibrinogen) from a two-component tissue sealant (Tisseel VH, Baxter, Westlake village, CA). Immediately after the second application, the thrombin component was added and particles of minced bladder mucosa were applied evenly and randomly orientated. Two pigs underwent sham procedure with same size excision of bladder tissue and thereafter implantation of the same type of tubing covered with fibrin glue but without minced particles.

Each tube was placed in the bladder with a purse string suture around the end of the tube (3 cm right angle part inside). The tube was placed tangential through the abdominal wall, thus penetrating the abdominal rectus muscle and the oblique muscles before placement of the other end in the subcutaneous tissue, immediately under the skin, and sutured with 4-0 nylon to stay in place (Ethilon, Ethicon, Johnson & Johnson, Sweden) (Figures 1(a) and 1(h)). Thereafter, the wall of the urinary bladder and the abdomen were repaired. The subcutaneous tissue was closed in two layers with absorbable braided sutures (Polysorb, Tyco Healthcare, Mansfield, MA) and the skin with interrupted 3-0 nylon sutures (Ethilon, Ethicon, Johnson & Johnson, Sweden). 

### 2.4. Catheterization and Radiological Assessment of 3D Conduit

At 4 and 5 weeks after transplantation, two pigs (one each week) were sedated with sodium pentothal (Pentobarbitalnatrium vet. 60 mg/mL, APL, Sweden) and fentanyl (Fentanyl vet. 50 *μ*g/mL, Braun, Germany) and placed in scanning equipment for angiography (XD20, Philips Medical System, Netherlands). In order to analyze the conduit for filling, emptying, and radiology, the transversal end of the tube, lying in the subcutaneous fat, was localized, skin was incised, and the end was cut off with scissors (Figures 1(a), 1(b), and 1(h)). The remaining tube was pushed into the bladder and the conduit was assessed from the newly created opening in the skin. An 8 Fr tube could be inserted through the conduit for filling and emptying the bladder. Intravenous contrast agent, iohexol (Omnipaque 240 mg/mL, GE Healthcare, Sweden) and diuretics furosemide (Furix 10 mg/mL, Takeda Pharma, Sweden) were given in order to visualize the urinary excretory pathway including the kidneys, ureters, the urinary bladder, and the conduit. 

**Figure 3:**
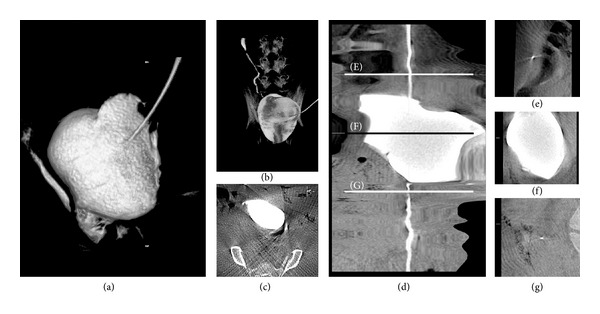
In order to visualize the anatomical properties of the conduit an XD20 imaging system was used. (a) 3D reconstruction showing the gross anatomical conditions. (b) The concomitant side projection. (c) A 0.5 mm thick CT reconstructed slice is shown. In order to illustrate the conduits passage to the bladder, a MultiPlanar (MP) reconstruction was performed through the CT stack illustrated in (d). Arrows in (d) indicate orthogonal projections along the MP reconstruction. In (e), (f), and (g) showing orthogonal projections along the MP reconstruction illustrating functioning ureters filling the bladder and intact conduit.

For radiological assessment of the conduit, reconstruction algorithm called Xper-CT, where the flat detector C-arm can perform volume acquisitions through back projection computed tomography (CT) reconstructions with a 0.5 mm slice thickness, was used [14]. Image stacks were then transferred to a working station, postprocessed and analyzed in Osirix v 4.1.1 software (Open Source, OsiriX Foundation, Geneva, Switzerland). 3D surface rendering was used for gross anatomical illustration after examining CT stacks in axial, frontal, and sagittal planes. To illustrate the entire tract including the intact conduit, 3D curved multiplanar reconstructions (MPR) were performed as illustrated in the figure (Figure 3(d)). Animals were euthanized after radiological assessments. 

### 2.5. Histological Examination

At 4 (6 animals) and 5 (2 animals) weeks posttransplantation, a lethal dose of sodium thiopental (Sodium Pentothal, Abbott Laboratories, North Chicago, IL) was administrated for termination. Tissue specimens including the whole conduit, from the subcutaneous tissue to the bladder were excised and fixed in 10% formalin solution prior to dehydration, embedding in paraffin and slicing into 5 *μ*m-thick sections. Slides were stained with haematoxylin-eosin (H&E) for routine histology, van Gieson for evaluation of collagen and other connective tissue, and periodic acid shift (PAS) for staining of carbohydrates in the basal lamina, connective tissue, and mucus, according to standard protocols.

The tissue specimens were evaluated by immunostaining with primary antibodies against various cytokeratins (1 : 300) (pancytokeratins 1–7, 10, 13–16 and 19, DakoCytomation, Cat# M0821, clone: MNF 116, Denmark) and uroplakin III (1 : 400) (Fitzgerald Industries, Clone AU1, CatRDI-PRO610108, Concord, MA). Biotinylated secondary antibodies were detected by using ABC-HRP kit (Vectastain Elite ABC kit, Vector Labs, UK) and all sections were developed employing 3,3′-diaminobenzidine (DAB) (Sigma Chemical Co., St. Louis, MO) substrate according to standard routine and manufacturer instructions. Mayer's hematoxylin solution was used for counter staining.

### 2.6. Measurements of Neo-Epithelialization

Mean, standard deviation, and full range were used to evaluate planimetric data from tissue sections. All measurements were performed under 100x magnification in a light microscope (Axioscope 2 MOT Zeiss microscope) using a built in ruler. We calculated the mean epithelialized area of 5 serial sections for each biopsy specimen at four different levels of each conduit; at the junction to the bladder (0 cm), 3 (in the abdominal muscle), 5 (between muscle and subcutaneous fat), and 8 cm (in the subcutaneous fat) from the junction. The percentage was calculated for each level by linear measurement of the epithelium in relation to the total inner luminal surface (18 Fr) (Figures 2(b)-2(c)). 

## 3. Results

All pigs tolerated the excision and removal of bladder tissue (approximately 1/4 of bladder) and voided shortly after surgery and none developed postoperative fever or signs of strain or pain when voiding. The first four animals were affected by midline hernia. Two had to be euthanized due to incarceration of the small intestine 2 and 4 days postoperatively. The surgical technique was changed for the next animals so that bladder conduit was placed from the side of the dome, to the left of the midline, through the rectus muscles, and not through the midline incision. One pig was euthanized the second day postoperatively due to signs of pain. Autopsy showed a perforated ulceration of the ventricle. The two surviving animals with midline hernia ful filled the study protocol and were euthanized four weeks after transplantation. In these subjects, the midline hernia including small intestines was encountered by the tube, no conduit had been formed and no epithelium could be detected in the surrounding tissue on histological examination. By these means, a total of five animals were excluded due to surgical complications.

Two of the animals were sedated and the 18 French latex tubes did not adhere to the adjacent tissue and could easily be pushed into the bladder (not removed through the skin due to the stopping end). The remaining conduit was easy to catheterize using 8 Fr catheters and lidocaine anesthetic gel (2% Xylocaine gel, AstraZeneca, Sweden) for lubrication. 

Xper-CT reconstructions clearly showed an intact conduit with contrast agent passage into the bladder both in the CT stacks reconstructed in three planes and in 3D surface rendered volumes (Figure 3). To illustrate the entire tract including the intact conduit 3D curved MPR, reconstructions were performed as illustrated in the figure (Figure 3(d)). This reconstruction clearly shows that the conduit is allowing fluid passage without leakage (Figures 3(a) and 3(b)). The bladder could be completely emptied by insertion of the catheter (not shown).

**Figure 4:**
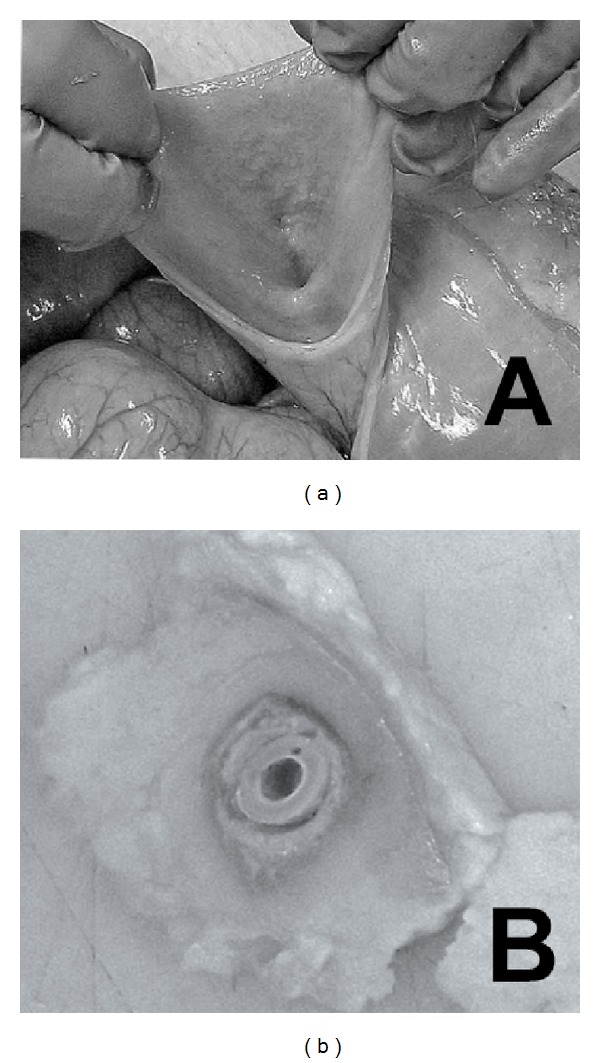
Macroscopic appearance at removal of conduit. (a) Looking from inside the bladder that has been cut open. Inner opening to conduit is exposed. (b) Macroscopic appearance of transsection of conduit with catheter still in place in the lumen.

Gross examination of the bladder revealed a scar in the frontal midline with no signs of inflammation. When opening the bladder, the mucosa had a normal appearance and the opening into the conduit revealed an orifice placed on the left side of the bladder dome with a normal mucosal lining (Figure 4(a)). Gross appearance of conduit revealed that the tube used for transplantation was lying loose inside the conduit and that the wall surrounding had an even inner tubular shape with a glossy surface under some nonadhering debris (Figure 4(b)).

**Figure 5:**
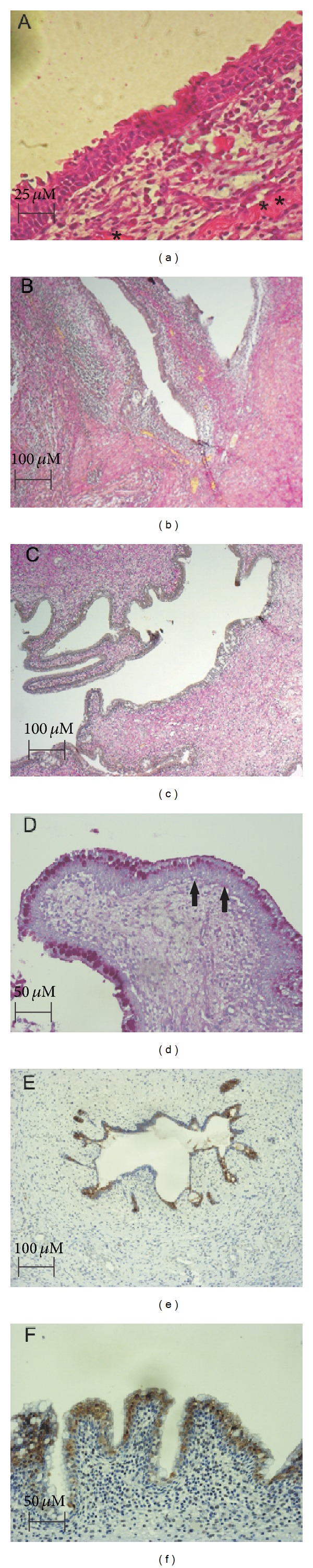
Microscopic analyses of tissue sections from conduit and from native bladder. (a) Wall of conduit covered with epithelium toward lumen after 4 weeks and submucosal capillaries (*) (H&E). (b) Staining of submucosal tissue from conduitwall (Van Gieson). (c) Staining of submucosal tissue from native pig bladder wall (Van Gieson). (d) Basal membrane under the epithelial cells (arrows) (PAS). (e) Immunostaining of cytokeratins (MNF116) in epithelial lining of conduit wall. (f) Immunostaining of Uroplakin III in epithelial lining of conduit wall.

Histological assessments were obtained at 4 (4 samples including one control) and 5 weeks (2 samples including one control) after transplantation (Table 1). Routine histology of full thickness biopsies of the wall of the constructed conduit demonstrated a continuous epithelium covering the inner surface of the conduit in all cases except controls (Figure 5(a)). Morphology revealed a transitional cell epithelium up to 4 or 5 cell layers thick. Submucosal tissue revealed signs of inflammation with infiltration of mononucleated cells and less mature extracellular matrix in comparison to bladder biopsies (Figures 5(b) and 5(c)). PAS reaction revealed a supporting basal lamina underlying the neoepithelium (Figure 5(d)). 

**Figure 6:**
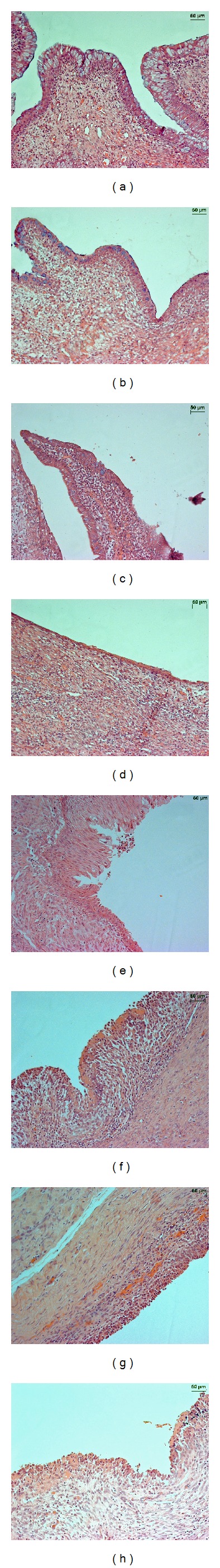
H and E staining of cross-sections from different parts of a conduit. (a)–(d): transplanted conduit. (e)–(h): sham operated control. (a) and (e) at junction between bladder and conduit. (b) and (f) 3 cm from junction. (c) and (g) 5 cm from junction. (d) and (h) 8 cm from junction.

Immunostaining of cytokeratins and uroplakin showed that the cells covering the inner lumen were of uroepithelial origin (Figures 5(e) and 5(f)). Histology at 4 and 5 weeks after transplantation revealed up to 90% uroepithelial lining but the area with epithelium varied between samples, and regenerated mucosa was more loosely adhering than in native bladder mucosa (Figures 6(a)–6(d)). The two specimens that were subjected to catheterization before histology revealed loose epithelium in the lumen. All samples (including controls) showed some urothelial cells at the junction to the bladder. At three and five centimeters from the junction, only transplanted conduits revealed epithelium (61, 22, 38, and 17%). At five centimeters, only half presented with epithelium (0, 0, 89, and 44%). From the most lateral part of the conduit, surrounded by subcutaneous fat, only traces of urothelial cells could be found in transplanted specimens. Conduit from control specimens had no epithelial lining in any tissue sections except at the junction to the bladder (Figures 6(e)–6(h)).

## 4. Discussion

In this porcine, large animal study, we have created catheterizable conduits by placing autologous urinary tissue around a three-dimensional mold (a tube) into the urinary bladder. The surgical technique is easy and fast forward and after removal of the mold, the conduit could be used for filling and emptying of the bladder. Advantages with the porcine model include a good-sized bladder for tissue harvesting and good tolerance for reduction of its size. No complications occurred in respect to infection.

We could document a patent conduit by administrating intravenous contrast and diuretics and performing 3D reconstructions of computed tomography images [14]. Kidneys excreted contrast and ureters and bladder were filled and then emptied through the tissue engineered bladder conduit. The use of 3D curved MPR was in accordance with CT urography guidelines used for high-definition visualization of an entire urinary tract albeit the use of angiographic equipment as primary imaging modality is not standard [15]. However, the Xper-CT algorithms give a high enough resolution to clearly provide an unambiguous image of the continuity of the open channel.

The rationale for transplanting small parts of minced tissue for epithelial cells expansion by using the patients own body as a bioreactor has been discussed in previous publications [12, 13, 16]. For this study, we used a similar expansion rate as described before for creating epithelium around tubular molds (approximately 1 : 3 expansion), however, under optimal conditions in a full-thickness wound healing model, a 100-fold expansion rate was successful after only 14 days [17]. This indicates that further improvements of the healing conditions in our experimental model could result in successful take and regeneration also with smaller amounts of tissue at initiation.

Due to the mincing device, particle size was basically uniform although for urothelium, it is sometimes hard to get 100% dissociation between the particles in comparison to minced skin epithelium. Sometimes 2–5 mucosa particles attach to each other even though they had been mechanically separated, the reason for this is probably the sticky glycosaminoglycans normally residing on the surface of the bladder mucosa [18]. Until now, we have not tried removing this mucous layer before mincing and transplantation. 

Histology at 4 and 5 weeks posttransplantation demonstrated a multilayered transitional uroepithelium along the inside of the newly formed conduit (Figure 5). The histology revealed that during this time period, the transplanted minced autologous urothelial transplants had reorganized from a random placement on the mold to a well-organized, continuous layer of mature epithelium facing the lumen and without adherence to the mold [13]. The basal membrane of the neo-epithelium covered the newly created channel. At the same time, tissue had expanded by division of cells from the transplanted tissue, most probably from the edges of the minced transplants according to the theory of Meek [19]. Histology from control animals showed no epithelium, which excludes that urothelium derived by migration from the bladder.

In this porcine *in vivo* study, we encountered difficulties that influenced the outcome negatively. Differences in pig anatomy, compared to human, include the intraperitoneal position of the urinary bladder. The animals had a risk of developing incarcerated intestinal midline hernias and the four animals that experienced this also had undesirable outcomes (two early terminations and two negative results). In this matter, we experienced a learning curve and by creating the conduit in a paramedian location through the abdominal rectus muscle, this problem was solved. Additionally, one pig was terminated due to acute peptic ulcer of unclear etiology. At the end of the study, a total of six conduits could be analyzed including two sham operated controls. 

Differences in body physiology including the resting and natural standing positions of the pig in combination with a fast increase in body weight (20 to 60 kg in 4 weeks) might also exert high-mechanical stress on the conduit [20]. The changes in histological appearance with inflammatory submucosal infiltration and loosely attached epithelium compared to native urothelium might be due to these factors.

In this study, we chose latex covered with a thin silicon elastomer to form the mold for guided tissue regeneration. The rationale for this was that previous studies showed a more favorable outcome in respect to neoepithelialization when compared to silicone molds [12]. After ten days *in vivo*, latex tubes demonstrated a significantly larger area of neoepithelialization in comparison to the silicon tube counterparts. As a proof of principle, we therefore chose latex in this animal model. However, as latex is not considered to be an acceptable material for implantation, in particular not in the group of patients that could potentially benefit from its application, we need to further study alternative materials in the molds before clinical application. Also, with longer time of exposure, 4-5 weeks, latex might have had a negative effect on the newly formed epithelium.

We also found that only half of the amount of conduits demonstrated urothelium at 5 cm from the bladder junction and only traces of urothelial cells were found at 8 cm (Table 1). One reason for this could be that the fatty tissue surrounding this part of the conduit had lower vascularization and poorer conditions for take of transplant than the medial part that passed through the abdominal muscles. The most lateral part might also be more susceptible for trauma by outer pressure to the skin as well as friction due to movements of the skin.

Histology of the two conduits that had been exposed to radiological examinations and catheterizations revealed urothelium in the lumen that had detached from the surface. The mechanical trauma from expelling the inner tube and insertion of a catheter most probably eroded the surface. This suggests that the neoepithelium is not strong enough for longstanding duration. In studies of transplanted cultured skin epithelium for treatment of severe burn injuries, the subepithelial tissue of the dermis seems imperative for good mechanical strength, stability, and to reduce the risk of peeling off and infection [21]. This might be a factor for bad attachment of the urothelium also in our cases. Although some submucosal tissue from the *lamina propria* is transplanted within the minced tissue, the regenerative properties of the cells and structures are not the same as for epithelium and thereby the supporting mechanical structures might not be strong enough in our study model.

In our previous studies using minced epithelium in the subcutaneous tissue, we also noted a tendency of loosening of the epithelial lining with time *in vivo *[12, 13]. Due to these findings, we hypothesized that contact with the bladder cavity and filling of urine would overcome the problem of placing a foreign body (the tube) in a blind cavity. However, as we still experience histological results with loosely adhering epithelium, we conclude that this mode of action was not enough for reducing the inflammatory reaction.

Although we found that minced urothelium expands and regenerates and that submucosal capillary networks develop that support epithelial nutrition, further improvements need to be undertaken in order for the newly formed tissue to withstand catheterizations several times a day. Most likely lack of appropriate *lamina propria* and a reduced amount of anchoring filaments and normal junctions between basal cells and the submucosa is the key problem for mechanical durability as the conduit runs through muscular and fatty tissue of the abdominal wall in analogy to problems related to transplanted cultured skin [22]. One mode of action might be to add minced bladder submucosal tissue with the minced bladder mucosa. We also hypothesize that a collagen carrier could support further submucosal tissue regeneration [23]. Preliminary studies have shown that a collagen-PCL hybrid construct does support migration, reorganization, and expansion of minced bladder mucosa *in vitro *and this could be next step for addressing this problem and further improving the method [24].

 In future studies, we would add a supportive matrix that could promote submucosal tissue regeneration and strengthen the anchorage of the neo-epithelial to the underlying tissue [16]. In a human setting, one of the advantages would be an extraperitoneal placement. Postoperatively, the patient would not be resting on the transplanted area and neither would a significant change of body weight be expected during the course of a month.

In conclusion, we demonstrate that it is possible to create conduits to the urinary bladder by randomly transplanting small particles of autologous urothelial tissue on a tubular mold placed in a surgically created canal through the abdominal wall. The method is easy to perform and the transplanted tissue specimens have capacity to expand around the mold without requiring any *ex vivo* cell culturing. Further studies are needed in order to evaluate the mechanical strength of the neo-epithelium in the newly formed conduits, in particular after injury that may occur when catheterizing the stoma. With further success, this could be of value for a large group of patients needing bladder emptying by catheterization through an abdominal stoma.

## Figures and Tables

**Table 1:** Table summarizing results from the analyses of conduits at different distances from the bladder junction. Animals numbered 1–4 were transplanted with minced urothelial particles, and animals 1 and 4 underwent radiological assessment before termination. At the junction, (0 cm) part of the native bladder was included in the biopsy. Biopsies from conduit running through the abdominal muscle (3 cm) reveal a median urothelial lining of 6 mm, SD 2.5 mm (33 ± 13%). Biopsy at the boarder between abdominal muscle and subcutaneous fat (5 cm) demonstrates a median urothelial lining of 6 mm, SD 6 mm (33 ± 33%).

Pig		Weeks after transplantation	0 cm (mm)	3 cm (mm)	3 cm (%)	5 cm (mm)	5 cm (%)	8 cm (mm)	Inner lumen (mm)
1	X-ray	4	18	11	61	+	+	+	18
2		4	18	4	22	+	+	+	18
3		4	18	6	33	17	89	+	18
4	X-ray	5	18	3	17	8	44	+	18
5	Control	4	16	0	0	0	0	0	18
6	Control	5	16	0	0	0	0	0	18

All transplanted conduits demonstrated some urothelial cells at the most distal part of the conduit but none in controls. Marking with (+) indicates the presence of urothelial cells but not as confluent epithelium at the inner luminal surface.
